# Meanings that mothers of obese children attribute to eating habits: grounded theory

**DOI:** 10.1590/1980-220X-REEUSP-2024-0330en

**Published:** 2025-05-12

**Authors:** Azucena Lizalde Hernández, María Mercedes Moreno González, Juliana Graciela Vestena Zillmer, Elizabeth Guzmán Ortíz, Josefina Valenzuela Gandarilla

**Affiliations:** 1 Universidad Michoacana de San Nicolás de Hidalgo, Morelia, Michoacán, Mexico.; 2 Universidad de Guanajuato, Salvatierra, Guanajuato, Mexico.; 3 Universidade Federal de Pelotas, Pelotas, RS, Brazil.

**Keywords:** Child Rearing, Caregivers, Feeding Behavior, Obesity, Qualitative Research, Educação Infantil, Cuidadores, Comportamento Alimentar, Obesidade, Pesquisa Qualitativa

## Abstract

**Objective:**

To describe the meanings that mothers of obese children attribute to eating habits in Morelia, Michoacán, Mexico.

**Method:**

Qualitative study, based on grounded theory and the premises of symbolic interactionism, conducted through semi-structured, individual, recorded and transcribed interviews, using intentional and theoretical sampling, with data analysis using the constant comparative method and the help of the ATLAS.ti software.

**Results:**

There were fourteen mothers as participants, with an average age of 36 years old, 50% in a stable union and 71% with paid work. The emerging categories were: 1. Mothers feeding based on their children's tastes, emotions and preferences; 2. Mothers compensating their children with food; 3. Mothers dealing with emotions; and 4. Mothers working and having to delegate childcare. Significant changes in eating habits were identified, since women, simultaneously exercising the roles of caregivers and providers, opt for quick and easy-to-prepare meals, which are most often ultra-processed food.

**Conclusions:**

The meaning attributed to eating habits emerges from the social interaction that the mother establishes with her children and her partner, being constructed based on tastes and food preferences and interpreted as an act of love and care.

## RESUMO

**Objetivo:**

Descrever os significados que as mães de crianças com obesidade atribuem aos hábitos alimentares em Morelia, Michoacán, México.

**Método:**

Estudo qualitativo, baseado na teoria fundamentada e nas premissas do interacionismo simbólico, realizado por meio de entrevistas semiestruturadas, individuais, gravadas e transcritas, utilizando amostragem intencional e teórica, com análise dos dados pelo método comparativo constante e o auxílio do *software* ATLAS.ti.

**Resultados:**

Participaram 14 mães, com idade média de 36 anos, sendo 50% em união livre e 71% com trabalho remunerado. As categorias emergentes foram: 1. Mães alimentando a partir dos gostos, emoções e preferências dos filhos; 2. Mães compensando os filhos com alimentos; 3. Mães lidando com emoções; e 4. Mães trabalhando e tendo que delegar o cuidado dos filhos. Foram identificadas mudanças significativas nos hábitos alimentares, uma vez que as mulheres, exercendo simultaneamente os papéis de cuidadoras e provedoras, optam por alimentos rápidos e fáceis de preparar, que na maioria das vezes são ultraprocessados.

**Conclusões:**

O significado atribuído aos hábitos alimentares emerge da interação social que a mãe estabelece com seus filhos e seu parceiro, sendo construído com base nos gostos e preferências alimentares e interpretado como um ato de amor e cuidado.

## INTRODUCTION

Eating habits are manifested in the way people act when selecting, preparing and consuming food, and are influenced by biological, psychological, social, economic, cultural and spiritual conditions in each context^(1,2,3)^. The United Nations has been working to eliminate all forms of malnutrition, aiming to ensure a healthy and well-being life for all by 2030, as well as to eradicate hunger, among other goals^(4)^. In this sense, each person’s diet should be complete, balanced, harmless, sufficient and varied^(1)^. However, not following these recommendations is quite common, we observe that people have been relying on a diet based on high-calorie foods, rich in saturated fats, salt and sugars^(3)^, which harms health by favoring the development of diabetes, heart disease, strokes, cancer^(4)^ and obesity – a condition characterized by excessive storage of fat in the body due to an imbalance between what is consumed and what is expended^(5)^. Therefore, having an adequate intake of vitamins and minerals should be everyone’s priority, so that the body can produce enzymes and hormones essential for growth and development. A healthy diet would save the lives of millions, in addition to avoiding serious repercussions on the health system and the global economy, since diseases resulting from an inadequate diet can cause millions of deaths and reduce productivity, compromising employability^(6)^. Globally, 155 million children are identified with delayed growth and development, and 41 million are overweight or obese^(5)^. According to the World Health Organization (WHO), obesity in children aged 5 to 19 is characterized by a Body Mass Index (BMI) for age greater than two standard deviations above the median of the growth reference, according to patterns that vary according to age group and gender^(5)^.

Some studies focused on variables, such as reduced BMI and weight loss, obtained temporary changes in behavior; Multimodal and multicomponent interventions, using information leaflets, workshops, games and other resources, have managed to increase knowledge about healthy eating, but without long-term results^(7,8)^. Other studies have evaluated parental practices related to monitoring the amount of food consumed by children, as well as the use of pressure, punishments, rewards and discipline^(9)^. The literature highlights the importance of parents in shaping their children’s eating habits, emphasizing that mothers play a central role in intervening in food preferences and eating styles^(9)^, and that it is their responsibility to offer and provide food from infancy to adulthood^(10)^.

In view of this, we identified a need to explore eating habits in depth based on the reality experienced, allowing a contextualized understanding, both at the individual level, as a unique human being, and in their family, cultural, religious, social, economic and political context. The objective was to describe the meanings that mothers of obese children attribute to eating habits in Morelia, Michoacán, Mexico.

## METHOD

### Study Design

This is a qualitative study, based on the theoretical perspective of symbolic interactionism^(11)^ and with a methodological reference in Grounded Theory, as proposed by Kathy Charmaz^(12)^.

### Participant Selection

This study was carried out through an initial intentional sampling, with the following inclusion criteria: mothers who prepared and shared at least one meal a day with their children and who had one or more children with obesity. The children’s body weight was measured in the selected elementary schools, using previously calibrated portable scales; the anthropometric measurement procedures were performed without shoes and with the children wearing light clothing^(13,14)^. Height was measured with a stadiometer equipped with a 2-m-long flexible metal measuring tape and a movable square forming a 90° angle, ensuring that the children were without shoes and without head ornaments or any other object that could interfere with the measurement^(13,14)^. Obesity was defined as BMI above the 95^th^ percentile, according to BMI tables for age and gender, using national health manuals for calculation (kg/m^2^). In addition to BMI, the age in months and gender (girl or boy) of the children were recorded, classifying the result as green for normal BMI, yellow for risk due to low BMI and red for danger due to high BMI^(15)^.

With the list of possible mothers, we initially contacted them through telephone calls, during which they were invited to participate in the study and informed of the data collection method, which was carried out by interview, with clarification of any doubts. After accepting, the participants were able to choose between several public places where they felt most comfortable or confident to conduct the interview: an elementary school classroom, home or public cafeteria, with the time scheduled according to each person’s availability.

### Data Collection

For data collection, an initial interview guide was prepared, structured after a literature review based on sensitizing concepts, and submitted to a prior pilot to verify its suitability. Data collection took place between March 2022 and March 2023, through intensive individual interviews, with face-to-face dialogue, conducted by the main researcher, who traveled to the location chosen by the participant in a quiet environment that allowed conversation.

The interviews were recorded with an electronic device and later transcribed for analysis^(16,17)^. To provide feedback on the data from the participants, an individual meeting was scheduled in an elementary school classroom, on a consensual date and time, during which the transcript was given so that each participant could read it and inform if they wished to add any additional information. On this occasion, the findings were presented through a diagram, and the properties of each category were explained, with the codes presented as pseudonyms of a flower and a fruit to ensure confidentiality^(12)^. Sampling was cumulative and sequential until data saturation was reached, identified when the information obtained stopped providing new aspects, implying the simultaneous collection and analysis^(12)^.

### Data Analysis

**Figure 1:**
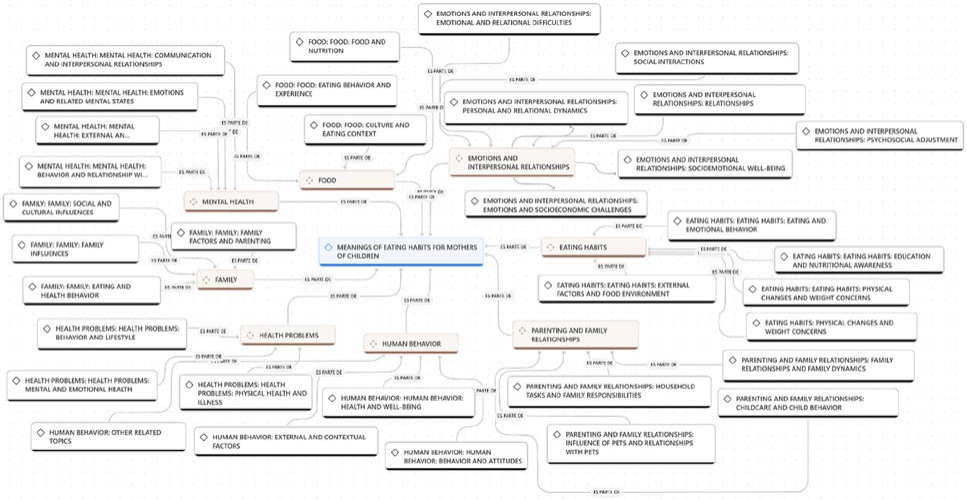
Coding Tree.

Source: ATLAS.ti Scientific Software Development GmbH version 23.

Data were coded inductively, beginning with independent coding of the first interview. Researchers then met to reflect on the data, creating initial codes, and other elements, using the Initial and Focused coding phases to describe the experience from each participant’s perspective. ATLAS.ti Scientific Software Development GmbH, version 23, was used to assist researchers in this process. The constant comparative method was applied, with researchers comparing data from one interview with data from subsequent interviews, as well as codes with codes, until the codes became more specific and conceptual, capable of explaining large segments of the data. Recurrent and significant codes were selected, which provided better analytical understanding to categorize the data or illuminate meanings about eating habits, elevating these codes to provisional theoretical categories that advanced focused coding. The most relevant codes were organized into subcategories and categories until the central category was identified. During this process, memos were prepared with analytical notes on the codes and ideas emerging from the data. The categories were grouped according to perceived similar attributes, allowing for emerging theoretical explanations and culminating in an abstract theoretical understanding of the studied experience (see Figure 1). The constant comparative method, based on the epistemological postulates of Grounded Theory, allowed the researchers to test ideas about what was observed, overcoming previous perspectives and identifying new elements that revealed meanings in a way that was adjusted to the data. The participants were removed from the field at the end of the analysis stage, at which point they were informed that their participation had concluded, and they were thanked for their collaboration and given general information about the preparation and delivery of the research results^(12)^.

### Rigor and Reflexivity

The following criteria of scientific rigor were applied: reflexivity, reliability, authenticity and transferability. From the beginning, beliefs, values and cultural and theoretical orientations were declared and disseminated, maintaining awareness of prior knowledge and personal and teaching assumptions. Reflexivity allowed the use of one’s own experiences to generate questions and deepen the analysis^(12,16,17,18,19)^. Before entering the field, the researchers carried out reflections, simultaneously and in light of the theoretical framework. Data collection and analysis occurred in parallel with the reflexivity process, with the aim of verifying the consistency of the data obtained and generating recommendations that reflected the codes evidenced in the meanings^(12,17,19)^.

### Ethical Considerations

This study was evaluated and approved by the Research Bioethics Committee under registration CONBIOÉTICA-16CEI-004-20161212 and by the Research Committee COFEPRIS-17-CI-16053153, registration 596/02/21. The participants were given the informed consent form, and an information sheet was provided. To ensure confidentiality, the children were assigned a pseudonym corresponding to the name of a fruit and the mothers were assigned the name of a vegetable. The transcripts and audio recordings are also kept securely by the researchers to ensure the protection of personal data^(20)^.

## RESULTS

### Sociodemographic Characteristics of Participants

Fourteen women participated in the study, with an average age of 36 years old; 50% reported they lived with their spouses, 71.4% were employed, and 51% relied on public social security for health care. The average age of their children was 10 years old (see Table 1). During data collection, three women chose not to participate, claiming that their children were already receiving medical care. In addition, two participants who initially agreed to participate decided to withdraw one day before the interview, as they were not allowed to arrive late for work, even after being offered another time.

**Table 1:** Sociodemographic characteristics of participants – Morelia, Michoacán, Mexico 2023.

Participants	Age	Maritul status	Educational level	Works	Social security	Children	Gender	Age	BMI
Margarida	27	Stable union	Incomplete Elementary I	No	None	Apple	M	9	32
Cravo	39	Stable union	Incomplete Elementary I	Yes	IMSS	Strawberry	M	11	26
Violeta	33	Divorced	Complete High School	Yes	IMSS	Banana	F	8	29
Gardênia	47	Divorced	Higher education	Yes	IMSS	Mango	F	9	28
Rosa	37	Stable union	Complete High School	Yes	None	Melon	F	7	27
Buganvília	27	Stable union	Ensino Fundamental II	Yes	None	Pear	F	6	24
Girassol	33	Stable union	Higher education	Yes	ISSSTE	Orange	F	10	23
Ave-do-paraíso	65	Married	Did not go to school	No	None	Ciriguela	F	10	42
Lavanda	35	Married	Elementary I	No	IMSS	Perón	M	10	23
Jasmim	35	Divorced	Elementary II	Yes	ISB	Blueberry	F	11	23
Hortência	31	Married	Elementary II	Yes	IMSS	Melon 2	M	10	23
Lírio	35	Married	Ensino Técnico	Yes	None	Melon 3	F	11	27
Dália	33	Stable union	Elementary II	No	IMSS	Grape 1	F	7	26
Orquídea	32	Stable union	Elementary II	Yes	None	Grape 2	F	8	33

Source: Participants’ data sheet.

Note: Mexican Social Security Institute (IMSS). Instituto de Seguridad y Servicios Sociales de los Trabajadores del Estado (ISSSTE). Instituto de Salud para el Bienestar (ISB).

The interviews lasted an average of 50.33 minutes. 953 codes emerged in the analysis process, from which, in its final stage, 4 main categories emerged:

### Category 1. Mothers Nurturing Based on Their Children’s Tastes, Emotions and Preferences

The taste for sweets is part of food preferences that, from the beginning of complementary feeding at 6 months, can become a very deep-rooted habit, difficult to control: *Because of work, I never made her cooked meals or any fruit… she usually ate the baby food that comes pre-packaged… but if I made her some homemade food, she would never eat it!* (Margarida). Milk is part of the customs for breakfast, but biscuits emerge as one of the sweet foods that children crave the most and that, in excess, becomes a problematic situation when it comes to nutritional balance: *He took a lot of cookies from the princesses for breakfast […] today he had milk and cookies* (Buganvília). Mothers end up giving in to their children’s demands to avoid crying, but at the same time, they recognize that they also do not want to harm their children and reflect on strategies in which they do not feel negative emotions such as guilt: *Until I said, no, enough! I made that mistake, so that he wouldn’t cry, I would give him a little bit more, and I said, no, no, what am I doing to my son? Enough! It doesn’t matter if he cries, but I’m not going to hurt him... I’m going to find another strategy so that I don’t feel guilty.* (Rosa).

### Category 2. Mothers Compensating Their Children With Food

Offering food is an act of giving love, a way of spoiling children with sweets and fried foods, even though mothers are aware that some of the foods they offer are not recommended: *They ask me, “Mommy, we want Nuggets!” Well, although I don’t like them that much, sometimes I spoil them; on Sundays, for example, they want cupcakes, but I try not to do it so often, because I feel happy, it’s a way of showing that I love them, I know that sometimes it’s not good, but I also love them.* (Dália). Feeling good about spoiling them with their favorite foods constitutes a pleasant sensation above rationality and leads mothers to act inappropriately when choosing the foods they offer their children: *It makes me feel good because they also make me feel like they like what I cook for them.* (Hortência).

Women make decisions to choose food for their husbands and children, from the union with their partner, these decisions involve her and her husband’s tastes, habits are formed in their new family: *when I`m with my husband, because they could afford [economic income] and his parents were always used to eating meat, so when I got there, I started eating more meat, which I didn’t eat when I lived with my parents and, because of that, I started offering it to my children* (Buganvília).

### Category 3. Mothers Dealing with Emotions

For some mothers, stress is a way of coping with everyday life; in some cases, by organizing their activities around feeding: *Since the evening I’ve been trying to leave something, so I’m not rushing... I feel like he eats slowly... in fact, I started getting more attention... I don’t know if it’s because I also eat very quickly, because of my work* (Violeta). Mothers’ childhood experiences with physical or emotional health problems can be significant today: *I am a person who`s traumatized by my body, I can admit it... oh, I won’t eat this anymore, I won’t eat that anymore!... I feel like it was also my problem that, perhaps, I didn’t ask my mother for help when I was a girl* (Cravo).

### Category 4. Working Mothers Having to Delegate the Care of Their Children

Family caregivers have been very significant in raising children, and among them, family caregivers stand out; the grandmother, for example, gains a lot of importance: *My mother has taken care of me my whole life; she takes care of children, I never put them in daycare* (Gardênia). Delegating childcare is a necessity for women, which makes it easier for them to go out to work; meal times are adjusted according to the caregivers’ ways of organizing themselves, which is difficult for mothers to control: *I go to work in the afternoon and he stays with his grandmother... he eats later, like at 5, that’s what I said... that, if I left him some chicken broth, he ate chicken and, then, at 8, he goes back to eating cereal or chocolate, that’s what he eats throughout the day* (Orquídea).

Mothers compensating with food expresses how mothers communicate with their children through emotions, showing love by being happy to feed them their favorite foods, although they feel obligation and concern for what they offer, recognizing that it is not always healthy and experiencing guilt. Mothers dealing with emotions reflects the reality of women who, as unique beings, face stress, low self-esteem, depression and other untreated emotional manifestations in their daily lives, which are normalized in interactions in social, family, work and other contexts. In turn, working mothers who have to delegate the care of their children emerge from the reality of those who have to entrust the care of their children to third parties in order to meet their work schedules, a situation that relates to all categories and occupies a central position in the model, due to its close connection. Furthermore, this factor constitutes an important social determinant in the meanings that mothers attribute to their eating habits when they go out to work.

Propositions were developed in the form of sentences that summarize ideas, observations and experiences. Thus, a proposition constituted a statement about a category and the relationship between two or more categories, while a non-relational proposition consisted of the definition of a concept. In this way, the concepts were considered as simple words or phrases, extracted and generalized by the researcher, summarizing ideas, observations and experiences^(12,18)^.

1. The food that mothers offer their children is an act of love and satisfaction in raising and controlling crying and tantrums to avoid confrontations with the child, while trying to control the consumption of unhealthy foods.
2. The meaning of mothers’ eating habits emerges from the social interaction they have with symbolic people such as their children and husband – they feed as an act of care, despite the concern about the physical and emotional consequences that this may have.
3. The meaning of eating habits changes when mothers feel uncertain about what their children eat when they delegate the care of feeding to caregivers, as they have to go out to work, a situation that is difficult for them to control; since they are faced with circumstances such as work schedules, domestic activities, stress, overload, etc.

## DISCUSSION

Propositions were developed in the form of sentences that summarized ideas, observations, and experiences. Thus, a proposition consisted of a statement about a category and the relationship between two or more categories, while a non-relational proposition represented the definition of a concept. Thus, it was considered that concepts could be simple words or phrases, extracted and generalized by the researcher to summarize ideas, observations, and experiences^(12,18)^.

The theoretical model was structured in interrelated categories, configuring a symbolic representation of the participants’ reality. This basis supports the beginning of the development of a substantial theory, capable of revealing interconnected phenomena. Categories from statements that clarify who, where, when, why, how, and with what consequences a phenomenon occurs were systematically integrated (Figure 1)^(18,21)^.

Blumer emphasizes that meanings develop and change over time. In this sense, women’s behavior, as wives and mothers, evolves and shapes eating habits through a social process that, according to their life trajectory, transforms cooking practices. In addition to being the main caregivers for their children, they are also responsible for their husbands’ well-being, playing a social and cultural role that, even in the face of economic and labor changes, remains the predominant activity for most: buying, preparing, and providing food for family members^(11,22)^.

During feeding practices, mothers face situations in which they give in to their children’s favorite foods. They interpret words, gestures, and attitudes of refusal toward vegetables and choose not to offer them, since they are not consumed. This decision arises from the tension between giving in and controlling, culminating in the offering of unhealthy foods, motivated by the concern that their children will not eat properly^(23)^. On the other hand, another study observed that mothers worry when their children do not eat, but do not want them to go hungry^(24)^. In this context, this concern can turn into a negative emotion, leading them to give in to their children’s favorite foods, even if they are unhealthy^(25)^. Similarly, some parents sought to offer their children everything they could – including unhealthy foods – to prevent them from experiencing hunger or restrictions^(23)^, always keeping options available that meet their children’s desires^(26)^.

Mothers and children interact emotionally through love, protection and overprotection in upbringing, and temperaments influence the decisions of mothers, who, driven by feelings of guilt and fear, give in out of love. Maternal concern is notorious, which intertwines with emotions such as stress and guilt, negatively influencing discipline, control or limitation of certain foods. By offering certain foods as a demonstration of affection, mothers feel less guilty. The lack of discipline may be related to the attempt to meet emotional needs through food^(25)^. In contrast, a study revealed that some parents negotiated or explained the amount of food allowed to their children, also offering healthier alternatives. The recognition of having contributed to poor eating habits – by not having offered certain foods to their children since childhood^(24)^ – reflects the influence of past experiences that negatively affect current habits^(23)^. Controlling negative emotions, a fundamental element for maternal care, is essential to be considered before starting intervention projects, ensuring adherence to treatment and the success of interventions^(27)^.

Self-control over emotional problems is essential for a holistic care in multidisciplinary collaboration, reflected in the fact that children end up eating the same things as their parents – who are responsible for teaching them to adopt a healthy diet and avoid contact with junk food^(26)^. In this way, mothers can prepare food according to their children’s health preferences, ensuring that they consume and enjoy it. In the interaction between the technological environment and eating practices, there are situations in which children eat in their rooms while watching television, a common practice for some families; on the other hand, other mothers fight for their children to eat at the table, adopting a more disciplined style^(22)^. Some parents forbid their children from eating on the sofa or in their rooms, as this implies associating mealtime with watching television, for example.

A study highlighted the importance of parents’ role in controlling television use: they are responsible for turning it on and off, instructing their children to only watch it when they respect the established rules – otherwise, they are punished by not watching it, and they also decide which programs they can watch^(26)^. The peace of mind parents feel when they know their children are eating “something” is a decision influenced by the concern that reduces discipline and control over permission to watch television while eating. Furthermore, due to interaction with a symbolic environment of greater availability of ultra-processed foods, women have adapted their eating habits^(28,29)^. In addition, it has been shown that technology influences the consumption of high-calorie foods^(25)^.

Mothers work and need to adjust their food preparation and consumption times according to their work schedules, preparing quick and easy food, although it is not healthy^(23)^. In another study, as a result of overwork, mothers ended up buying pre-prepared food^(25)^. There are also coincidences regarding the restriction of time to prepare food^(30)^. Other studies have found that parents’ overwork prevents them from preparing healthy foods and, due to lack of time, they have to buy ready-made food outside the home^(25)^, which also adds to their preferences and taste. In addition, it is necessary to delegate childcare to other family caregivers, with whom mothers share the care of their children, especially during working hours. It is in this process that children share food with caregivers, and food-rearing practices influence habits^(23,29)^. The above highlights the need for interventions that involve caregivers to promote eating habits from the family.

**Figure 2:**
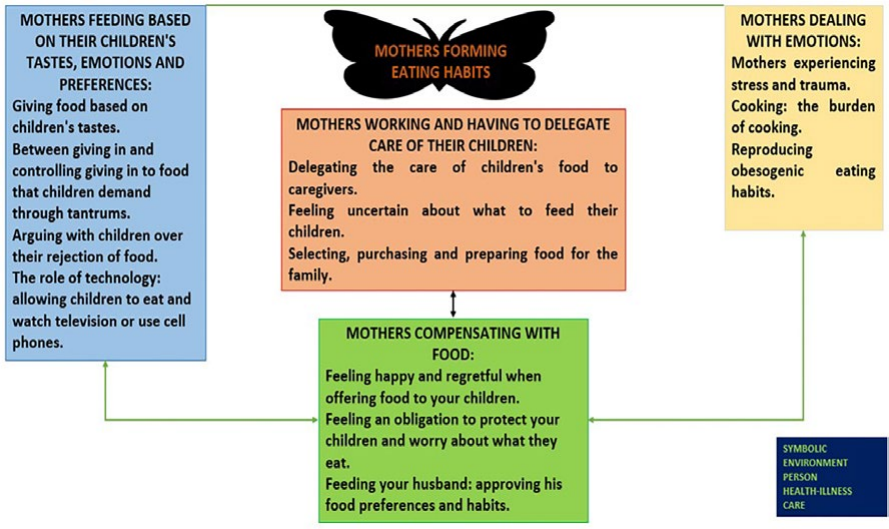
Model of the meanings that mothers of obese children attribute to eating habits.

Source: Own creation, 2023.

Moving towards new healthy eating habits, compatible with the economic resources and culturally acceptable in each family, filling gaps through public policies for female heads of households, zero hunger, health and well-being for women and children. Finally, families have symbolic people such as the husband; The findings of this study show the mother’s interaction with her husband’s food preferences and tastes and the strong influence on the meanings that women attribute to what they buy and cook to feed all family members. This is evident in the testimonies, showing the father’s authority and the women’s lack of authority; in addition, the father’s beliefs about a magical change in the children’s weight — that he will resolve it when the children grow up, without recognizing obesity as a disease — (Figure 2)^(25)^.

## STUDY LIMITATIONS

Due to the variability in the organizational systems of educational institutions and the differences in class and vacation dates, data collection and analysis were interrupted. Although the breaks were considered suspensions and vacations, it was difficult to follow up on the interviews during the periods of internal suspension. Identifying sensitizing concepts and writing reflective memos about the researchers’ personal and professional experiences helped to minimize bias, allowing them to acknowledge their own beliefs, knowledge, experiences, emotions and feelings, which transformed these conditions into an opportunity to deepen the conversations.

## CONCLUSION

Women, as social and holistic beings, play multiple roles assigned by society, such as being a woman, wife and mother. However, socially, economically and culturally, they have evolved, starting to work and taking on responsibilities in composing their children’s diet. Feeling guilty about the type of food they offer, mothers tend to give in – because that is what their children eat – in order to avoid crying and irritation, continuing to try to control the consumption of what they recognize as unhealthy, but, on the other hand, resorting to imposing vegetables that their children reject.

The Meanings model, attributed to mothers of obese children, proposes valuing women as social beings and constitutes a tool for recognizing their integrality as members of the family nucleus. In this context, nursing care enables the expression and recognition of their feelings, emotions and concerns. The testimonies highlight the complexity of the realities experienced by women who, faced with altered emotional responses – recognized as normal –, require individualized care plans that take into account their food preferences, culture and particularities. Thus, family care planning advances towards a humanized approach, which recognizes meanings as social constructions inserted in the family, social and cultural context.

A change in the cultural paradigm in care is imperative, encouraging the integration and mandatory participation of men in interventions, due to their important contribution in shaping the eating habits of both women and children. Although the roles of men and women have evolved – with both parents now working and contributing to the economy –, activities related to food preparation and other tasks traditionally assigned to women need to be reviewed in order to alleviate the burden on these professionals, who, in addition to working, accumulate household chores and childcare.

According to the women’s perception, their partners’ eating habits and attitudes strongly influence the formation of their children’s habits and behaviors. In this scenario, regulating tastes and meeting the husband’s food preferences opens up an opportunity for nursing care by recognizing the cultural and social relevance of the father’s role as a husband. Therefore, it is essential that fathers are informed and aware of the importance of consuming and offering a varied, balanced and healthy diet, which will have a positive impact on their health and that of their children throughout their lives, promoting well-being and quality of life.
