# Supplementary material for: Meanings that mothers of obese children attribute to eating habits: grounded theory
Source: Rev Esc Enferm USP. 2025 May 12;59:e20240330. doi: 10.1590/1980-220X-REEUSP-2024-0330en (PMC12070806; doi:10.1590/1980-220X-REEUSP-2024-0330en)
Supplement: Translation

# Translation: Significados que madres de niños con obesidad tienen sobre los hábitos alimentarios: teoría fundamentada

**DOI:** 10.1590/1980-220X-REEUSP-2024-0330es

**Authors:** Azucena Lizalde Hernández, María Mercedes Moreno González, Juliana Graciela Vestena Zillmer, Elizabeth Guzmán Ortíz, Josefina Valenzuela Gandarilla

**Affiliations:** 1 Universidad Michoacana de San Nicolás de Hidalgo, Morelia, Michoacán, Mexico.; 2 Universidad de Guanajuato, Salvatierra, Guanajuato, Mexico.; 3 Universidade Federal de Pelotas, Pelotas, RS, Brazil.

**Keywords:** Crianza del Niño, Cuidadores, Conducta Alimentaria, Obesidad, Investigación Cualitativa

## Abstract

**Objetivo:**

Describir los significados que las madres de niños con obesidad tienen sobre los hábitos alimentarios en Morelia, Michoacán, México.

**Método:**

Estudio cualitativo, teoría fundamentada, premisas del interaccionismo simbólico. Se realizaron entrevistas semiestructuradas, individuales grabadas y transcritas con muestreo intencional y teórico. Análisis de datos, método comparativo constante, software ATLAS. Ti.

**Resultados:**

Participaron 14 madres, con edad media de 36 años, unión libre (50%) y trabajo remunerado (71%). Categorías emergentes: 1. Madres alimentando a partir de los gustos, emociones y preferencias de los hijos, 2. Madres compensando a hijos con alimentos, 3. Madres lidiando con emociones, 4. Madres trabajando y teniendo que delegar el cuidado de sus hijos. Se identificaron cambios significativos en los hábitos de alimentación; las mujeres al ser cuidadoras y proveedoras de manera simultánea eligen alimentos rápidos y fáciles de preparar que generalmente son ultra procesados.

**Conclusiones:**

El significado sobre los hábitos alimentarios emerge de la interacción social que la madre mantiene con sus hijos y su pareja; se construyen de acuerdo con los gustos y preferencias alimentarias; son atribuidos como un acto de amor y cuidado.

## INTRODUCCIÓN

Los hábitos alimentarios se manifiestan en las formas de actuar de las personas cuando seleccionan, preparan y consumen alimentos; influenciados por condiciones biológicas personales, psicológicas, sociales, económicas, culturales y espirituales en cada contexto^(1–3)^.

Las Naciones Unidas, trabajan para eliminar la malnutrición en todas sus formas y esperan que para el año 2030 se garantice para todos, una vida sana, con bienestar, poner fin al hambre, entre otros^(4)^. La dieta de cada persona deberá ser completa, equilibrada, inocua, suficiente y variada^(1)^. No obstante, en muchas ocasiones no se cumple con dichas recomendaciones, dado que se identifica una alimentación a base de alimentos hipercalóricos ricos en grasas saturadas, sal y azúcares^(3)^, lo que perjudica el estado de salud, desde el desarrollo de la diabetes, cardiopatías, accidentes cerebrovasculares y cáncer^(4)^, así como la obesidad que se caracteriza por almacenar grandes cantidades de grasa en el cuerpo al enfrentar un desequilibrio energético entre lo que comen y gastan^(5)^.

Por lo que es prioritario que todas las personas tengan una ingesta suficiente de vitaminas y minerales para que el organismo produzca enzimas y hormonas esenciales para un crecimiento y desarrollo adecuado. Una alimentación saludable salvaría la vida de millones de personas; además de evitar serias repercusiones en el sistema de salud y en la economía mundial; las enfermedades por alimentación no adecuada pueden causar millones de muertes, las personas enfermas serán poco productivas y con menos probabilidades de estar empleadas^(6)^. En todo el mundo se identifican 155 millones de niños con retraso en crecimiento y desarrollo, 41 millones tienen sobrepeso u obesidad^(5)^. De acuerdo con la Organización Mundial de la Salud (OMS) la obesidad en niños de 5 a 19 años corresponde a un Índice de Masa Corporal (IMC) para la edad superior a dos desviaciones típicas por encima de la mediana de la referencia de crecimiento, se determinan mediante patrones de crecimiento infantil, que va de acuerdo con su grupo de edad y sexo^(5)^.

Algunos estudios centrados en variables como la disminución en el IMC y la pérdida de peso obtuvieron cambios de conducta temporales; las intervenciones multimodales y multicomponente que, a través de folletos informativos, talleres, juegos y otros, lograron incrementar el conocimiento relacionado con la alimentación saludable, pero sin resultados a largo plazo^(7,8)^. Otras investigaciones, han evaluado las prácticas parentales sobre la supervisión de la cantidad de alimento que comen los hijos, la presión, castigos, recompensa y disciplina^(9)^.

La literatura establece que los padres juegan un rol importante en los hábitos alimentarios de los hijos, principalmente la madre interviene en las preferencias alimentarias y los estilos de alimentación^(9)^. El desempeño de la madre al ofrecer y otorgar alimentos a los hijos es parte de su responsabilidad desde la infancia hasta que cumplan la mayoría de edad^(10)^.

Por lo anterior se identificó la necesidad de explorar a profundidad los hábitos alimentarios a partir de su realidad que permita la comprensión en un contexto particular, en lo individual como ser humano único y en su contexto familiar, cultural, religioso, social, económico y político. El objetivo fue describir los significados que las madres de niños con obesidad tienen sobre los hábitos alimentarios en Morelia, Michoacán, México.

## MÉTODO

### Diseño de Estudio

Estudio cualitativo, perspectiva teórica del interaccionismo simbólico^(11)^, referente metodológico Teoría Fundamentada en los datos propuesta por Kathy Charmaz^(12)^.

### Selección de los Participantes

La búsqueda de las participantes se realizó a través de muestreo inicial intencional, criterios de inclusión: madres que prepararan y compartieran al menos una comida del día con su hijo, tener uno o más hijos con obesidad.

La medición del peso corporal de los niños se llevó a cabo en las escuelas primarias seleccionadas; se emplearon básculas portátiles previamente calibradas, la medición antropométrica se realizó sin zapatos y con ropa ligera^(13,14)^. La medición de la talla se realizó con un estadímetro provisto de cinta métrica de metal flexible con 2m de longitud y escuadra móvil con un ángulo de 90°, los niños se colocaron sin zapatos, ni adornos en la cabeza o cualquier otro objeto que interfiriera con la medición^(13,14)^. Se determinó la obesidad cuando el IMC se encontró desde el percentil 95 en adelante, de las tablas de IMC para edad y sexo; se utilizaron las cartillas nacionales de salud para calcular el IMC (kg/m^2^). Se valoró de acuerdo con el resultado de IMC la edad en meses, si era niña o niño y se ubicó el en color verde como IMC normal, amarillo riesgo por IMC bajo y roja peligro por IMC elevado^(15)^.

Con la lista de posibles madres, se contactó por llamada telefónica, se invitó a participar y explicó el método de recolección de datos a través de entrevista y dilucidó dudas. Tras la aceptación, se dieron opciones de diversos lugares públicos a las participantes para elegir donde se sintieran cómodas o con más confianza: 1. Aula de escuela primaria. 2. Vivienda, 3. Cafetería pública; el horario fue establecido por la participante de acuerdo con su disponibilidad.

### Colecta de Información

Para la recopilación de datos se elaboró una guía de entrevista inicial, la cual se estructuró después de una revisión de la literatura a partir de conceptos sensibilizadores; se realizó pilotaje previo con la finalidad de comprobar la adecuación de la misma. La obtención de datos ocurrió en los meses de marzo 2022 a marzo de 2023, se llevó a cabo una entrevista individual intensiva por participante, con un diálogo cara a cara, realizada por la investigadora principal, quién acudió al lugar elegido por la participante y se verificó un ambiente tranquilo y que permitiera el diálogo. Las conversaciones fueron grabadas con ayuda de un dispositivo electrónico y posteriormente transcritas para su análisis^(16,17)^. Para la retroalimentación de las participantes sobre los datos, se agendó de manera individual una reunión en aula de escuela primaria, previo consenso de fecha y hora, durante la reunión se entregó la transcripción para que cada una estuviera en posibilidad de leer, además se preguntó si deseaban agregar algún otro dato; igualmente, se presentaron los hallazgos en el diagrama y de cada categoría se explicaron las propiedades, se mostraron códigos vivos, todos con pseudónimos de una flor y fruta para garantizar la confidencialidad^(12)^. El muestreo fue acumulativo y secuencial hasta llegar a la saturación de datos, identificado cuando la información obtenida no aportaba aspectos nuevos, lo que implicó la realización simultánea de recolección y análisis^(12)^.

### Análisis de los Datos

Los datos se codificaron inductivamente, la primera entrevista fue codificada de manera independiente, posteriormente se reunieron para reflexionar sobre los datos, códigos iniciales, entre otros; utilizaron las siguientes fases de codificación: Inicial y enfocada; para describir la experiencia desde la perspectiva de lo que cada participante ha experimentado, se utilizó el ATLAS.ti Scientific Software Development GmbH versión 23.

El análisis comparativo constante realizado por cada uno de los investigadores, comparando datos de una entrevista con datos de entrevistas subsecuentes, códigos con códigos; hasta que los códigos fueron más específicos y conceptuales que explicaron grandes segmentos de datos, se eligieron los códigos recurrentes, significativos que daban una mejor comprensión analítica para categorizar los datos o para iluminar los significados sobre los hábitos alimentarios elevando esos códigos a categorías teóricas provisionales que avanzaron la codificación enfocada, los códigos anteriores más significativos se organizaron en subcategorías y categorías hasta llegar a la categoría central^(12-17)^. Durante el proceso se redactaron memorándums con notas analíticas sobre los códigos e ideas sobre los datos^(14)^. Las categorías se agruparon con atributos similares percibidos para explicar lo que estaba sucediendo en los datos permitiendo emplearse en la explicación teórica emergente; hasta lograr la comprensión teórica abstracta de la experiencia estudiada (ver Figura 1)^(12–18)^. El análisis comparativo constante, parte de los postulados epistemológicos de la Teoría Fundamentada, permitió a las investigadoras probar las ideas sobre lo que se veía en los datos más allá de las perspectivas previas, permitiendo identificar lo nuevo o novedoso y sacar a la luz los significados utilizando términos que se ajustaron a los datos^(12)^. La retirada del campo se realizó al concluir la etapa de análisis, se informó que su participación en el presente estudio había concluido agradeciendo su colaboración e informando de manera general los elementos para la elaboración y entrega de los resultados de la investigación^(12)^.

**Figura 1:** Árbol de codificación.

Fuente: ATLAS.ti Scientific Software Development GmbH versión 23.

### Rigor y Reflexividad

Se aplicaron los criterios de rigor científico: reflexividad, fiabilidad, autenticidad y transferibilidad; al inicio se declararon y dieron a conocer las creencias, valores, orientación cultural y teórica, se permaneció consciente de conocimientos previos, las presuposiciones personales y docentes; también, la reflexividad permitió tomar la propia experiencia para generar preguntas y profundizar en el análisis^(12,16–19)^. Previo a la entrada al campo se realizaron reflexiones entre las investigadoras a la luz de referente teórico, de manera simultánea se realizó la colecta y análisis de los datos de la mano del proceso de reflexividad con el propósito de verificar la consistencia de los datos obtenidos, generar recomendaciones para reflexionar los códigos que pusieran en manifiesto los significados^(12,17,19)^.

### Consideraciones Éticas

El presente estudio fue evaluado y aprobado por el Comité de bioética en Investigación con registro CONBI-OÉTICA-16CEI-004-20161212 y el Comité de Investigación COFEPRIS-17-CI-16053153, registro 596/02/21. Se obtuvo la firma de consentimiento informado, asentimiento y se entregó hoja de información, a los niños se les asignó un pseudónimo con el nombre de una fruta y a las madres el nombre de una verdura lo que permitió garantizar su confidencialidad. La transcripción y los audios se mantienen en resguardo por los investigadores para garantizar la protección de datos personales^(20)^.

## RESULTADOS

### Características Sociodemográficas de las Participantes

Participaron en el estudio 14 mujeres con una edad media de 36 años, el 50% de las participantes refiere vivir en unión libre; el 71.4% trabajan y el 51% cuenta con seguridad social pública para la atención de la salud. La edad media de los hijos fue de 10 años (Ver Tabla 1). Durante la colecta de datos tres participantes no aceptaron participar refiriendo que sus hijos ya estaban en atención médica. Dos participantes que sí aceptaron participar, un día antes de la entrevista decidieron retirarse porque en su trabajo no les autorizaron llegar más tarde y aunque se ofreció otro horario no aceptaron.

**Tabla 1:** Características sociodemográficas de las participantes – Morelia, Michoacán, México 2023.

Participantes	Edad	Estado civil	Nivel educativo	Trabaja	Seguridad social	Hijo	Género	Edad	IMC
Margarita	27	Unión libre	Primaria incompleta	No	Ninguna	Manzana	M	9	32
Clavel	39	Unión libre	Primaria incompleta	Si	IMSS	Fresa	M	11	26
Violeta	33	Separada	Preparatoria completa	Si	IMSS	Banana	F	8	29
Gardenia	47	Separada	Licenciatura	Si	IMSS	Mango	F	9	28
Rosa	37	Unión libre	Preparatoria completa	Si	Ninguna	Melón	F	7	27
Buganvilia	27	Unión libre	Secundaria terminada	Si	Ninguna	Pera	F	6	24
Girasol	33	Unión libre	Licenciatura terminada	Si	ISSSTE	Naranja	F	10	23
Ave del paraíso	65	Casada	No estudio	No	Ninguna	Ciruela	F	10	42
Lavanda	35	Casada	Primaria terminada	No	IMSS	Perón	M	10	23
Jazmín	35	Separada	Secundaria terminada	Si	ISB	Blueberry	F	11	23
Hortensia	31	Casada	Secundaria terminada	Si	IMSS	Melón2	M	10	23
Lirio	35	Casada	Carrera técnica	Si	Ninguna	Melón3	F	11	27
Dalia	33	Unión libre	Secundaria terminada	No	IMSS	Uva1	F	7	26
Orquídea	32	Unión libre	Secundaria terminada	Si	Ninguna	Uva2	F	8	33

Fuente: Cédula de datos de participantes.

Nota: Instituto Mexicano del Seguro Social (IMSS). Instituto de Seguridad y Servicios Sociales de los Trabajadores del Estado (ISSSTE). Instituto de Salud para el Bienestar (ISB).

Las entrevistas tuvieron una duración media de 50.33 minutos; surgieron 953 códigos del proceso de análisis, del cual en su etapa final emergieron 4 categorías principales:

### Categoría 1. Madres Alimentando a Partir de los Gustos, Emociones y Preferencias de los Hijos

El gusto por el dulce hace parte de las preferencias alimentarias que, desde el inicio de la alimentación complementaria a los 6 meses de edad, se puede convertir en una costumbre muy arraigada difícil de controlar: *A ella por lo mismo del trabajo yo nunca le hice un chayote hervido o alguna fruta… ella normalmente lo que comía eran las papillas que ya vienen empacadas… pero así de yo hacerle alguna casera no ¡nunca!* (Margarita). La leche hace parte de las costumbres en el desayuno, pero, las galletas emergen como uno de los alimentos dulces que los niños más apetecen y que, su exceso se convierte en una situación problemática cuando se trata de balance nutricional: *Agarró por desayunar muchas galletas de las príncipes […] hoy tomó leche y galletas (*Buganvilia). Las madres terminan cediendo frente a las exigencias de los hijos para evitar el llanto, pero al mismo tiempo reconocen que tampoco quieren hacerle un mal y reflexionan sobre estrategias en las cuales ellas no sientan emociones negativas como la culpa: *hasta que yo dije ¡no, ya! yo caí en ese error, con tal de que no llorara, le daba otro poquito, y yo dije no, no, ¿qué estoy haciendo con mi niño? ¡ya! no importa que llore, pero no le voy a hacer un mal… voy a buscar otra estrategia donde yo no me sintiera culpable (Rosa).*

### Categoría 2. Madres Compensando a Hijos con Alimentos

Dar alimentos es un acto de dar amor, una forma de consentir a los hijos con dulces y fritos, a pesar de que las madres son conscientes de que algunos de esos alimentos que otorgan no son recomendables:: *me piden ¡mamá queremos Nuggets! pues no me gusta tanto, pero así también a veces sí los consiento, los domingos, por ejemplo, me piden los cupcakes, pero trato pues de que no sea tan seguido, pues yo me siento feliz, es una forma de demostrarles que los amo, ¡sé que a veces no es bueno! pero sí también lo amo (Dalia).* Sentirse bien al consentirlos con sus alimentos preferidos, constituye una sensación agradable por encima de la racionalidad y lleva a la madre a actuar en forma inadecuada ante la elección de los alimentos que otorgan a sus hijos: m*e hace sentir bien porque, también ellos me hacen sentir que les gusta lo que yo les cocino* (Hortensia).

Las mujeres toman decisiones para elegir alimentos para su esposo e hijos, a partir de la unión con su pareja en estas decisiones se involucran los gustos de ella y de su esposo, se forman hábitos en su nueva familia: *cuando yo me junté con mi esposo, pues tenían un poquito más [ingreso económico] sus papás siempre estuvieron acostumbrados a comer carnes, entonces yo, llegar allá y empecé a comer más carne, lo que no comía cuando vivía con mis papás y por eso empecé a metérselo a mis hijos:* (Buganvilia).

### Categoría 3. Madres Lidiando con Emociones

Para algunas madres el estrés constituye una forma de afrontar la vida diaria; en algunos casos mediante la organización de sus actividades para la alimentación: *desde la noche trato de dejarle algo, para no estar corriendo... siento que come como lento… la verdad, sí le empecé a llamar la atención … no sé si sea porque yo también como muy rápido, por lo de mi trabajo* (*Violeta).* Las experiencias en la infancia de las madres ante problemas de salud físicos o emocionales podrían ser significativas en la actualidad: *yo soy una persona traumada con mi cuerpo, lo puedo admitir… ay ya no voy a comer esto, ¡ya no voy a comer lo otro! … yo siento que también fue mi problema que a lo mejor no le pedí ayuda a mi mamá cuando yo era más niña* (Clavel).

### Categoría 4. Madres Trabajando y Teniendo que Delegar el Cuidado de sus Hijos

Los cuidadores familiares han sido muy significativos en la crianza de los niños, y, de ellos, se destacan los cuidadores familiares, la abuela, por ejemplo, cobra mucha importancia: m*i mamá toda, toda la vida me los ha cuidado; ella se queda con los niños, nunca los tuve en guardería* (Gardenia). Delegar el cuidado de los hijos es una necesidad de las mujeres, lo cual facilita las condiciones para que ellas salgan a trabajar, los horarios de alimentación son ajustados de acuerdo con las formas de organización de los cuidadores, lo que para las madres, es difícil de controlar: *yo me voy a trabajar en la tarde y se queda con su abuelita… come después, como a las 5, es lo que le dije yo… que si le dejé un caldo de pollo, se comió pollo y como eso de las 8, vuelve a comer cereal o un chocomil, es lo que comen en el transcurso del día* (Orquídea).

**Madres compensando con alimentos**, se manifiesta como las madres se comunican con sus hijos a través de emociones como muestra de amor porque sienten felicidad alimentarlos con lo que los hijos prefieren, sensación de obligación para alimentarlos a pesar de preocuparse por lo que les otorgan, reconociendo que no es saludables y sentir culpas. **Madres lidiando con emociones**, refleja la realidad de las mujeres como seres únicos que lidian en su cotidianeidad con el estrés, baja autoestima, depresión y otras manifestaciones emocionales no atendidas y normalizadas como parte de la interacción en entornos sociales, familiares, laborales y otros. **Madres trabajando y teniendo que delegar el cuidado de sus hijos**, emerge de la realidad de madres que tienen que delegar el cuidado de sus hijos para cumplir horarios laborales, se relaciona con todas las categorías y se muestra al centro del modelo por su estrecha relación; además, importante determinante social en los significados que madres atribuyen a los hábitos alimentarios al salir a trabajar.

Se desarrollaron proposiciones como frases que resumen ideas, observaciones y experiencias. Una proposición fue una declaración sobre una categoría y la relación entre dos o más categorías; una proposición no relacional fue una definición sobre un concepto; asimismo, se consideró que los conceptos podrían ser palabras o frases simples extraídas y generalizadas por la investigadora, frases que resumen ideas, observaciones y experiencias^(12,18)^.

1. La alimentación que ofrecen las madres hacia sus hijos significa un acto de e amor y satisfacción en la crianza y control del llanto y berrinches para evitar confrontación con el hijo quedándose en el intento de controlar el consumo de alimentos no saludables.
2. El significado de los hábitos alimentarios de las madres emerge de la interacción social que mantiene con personas simbólicas como sus hijos y esposo, se alimentan como acto de cuidado pese a la preocupación por las consecuencias físicas y emocionales que pudieran tener.
3. El significado de los hábitos de alimentación se modifica cuando las madres sienten incertidumbre sobre lo que sus hijos comen cuando delegan el cuidado alimentario a cuidadores, porque tienen que salir a trabajar, situación que es difícil para ellas controlar; ya que se enfrentan a circunstancias de horarios laborales, actividades en el hogar, estrés, sobrecarga, etc.

## DISCUSIÓN

El modelo teórico se estructuró en categorías que se interrelacionaron, esta representación simbólica de la realidad de las participantes es la base en el inicio del desarrollo de una teoría sustantiva que revela fenómenos relacionados entre sí; se integraron sistemáticamente categorías emanadas de enunciados que explicaron quién, dónde, cuándo, por qué, cómo y con qué consecuencias se produce un fenómeno (Figura 1)^(18,21)^. Blumer refiere que los significados se desarrollan y cambian a través del tiempo, en ese sentido el comportamiento de la mujer como esposa y madre evolucionan y forman hábitos alimentarios en un proceso social, que de acuerdo con su trayectoria de vida modifican las formas de cocinar; son cuidadoras principales no sólo de los niños, también del esposo, un rol social y cultural que a pesar de diversos cambios económicos y laborales es una actividad de la mayoría de las mujeres comprar, preparar alimentos y otorgar alimentación a los integrantes de la familia^(11,22)^.

Durante la práctica de alimentación, las madres lidian con situaciones, cediendo a los alimentos que sus hijos prefieren comer; ellas interpretan palabras, gestos y actitudes de negación hacia las verduras y deciden ya no darlas porque no comen, decisión que emerge de la tensión entre ceder y controlar para finalmente otorgar alimentos poco saludables, decisión que surge de su preocupación por que su hijo no come^(23)^, caso contrario, en otro estudio, las madres si se preocupan cuando sus hijos no comen pero ellas no quieren que sus hijos pasen hambre^(24)^, en esta dificultad, la preocupación puede ser una emoción negativa que influye para que las madres cedan los alimentos que los hijos prefieren a pesar de que estos no sean saludables^(25)^. Algunos padres trataron de dar a sus hijos todo lo que pudieron, entre ellos la comida no saludable y evitar que sus hijos no experimentaran hambre o restricciones^(23)^, tener comida siempre disponible a complacencia de los hijos^(26)^. Madres e hijos tienen formas de interacción de emociones como el amor, protección y sobreprotección en la crianza de hijos cuyos temperamentos influyen sobre las decisiones de las madres que con sentimientos de culpas y miedos ceden por amor. Es evidente la preocupación de las madres en conjunto a las diversas emociones como estrés y sentimientos de culpa que podrían ser emociones negativas en la disciplina, control o limitación hacia algunos alimentos, condiciones que afronta otorgando algunos alimentos como muestra de amor hacia sus hijos porque ellas se sienten bien con menos culpas. La falta de disciplina podría estar relacionada a complacer las emociones con alimentos^(25)^. En caso contrario, un estudio muestra que algunos padres negociaron o explicaron qué cantidad de alimento les permitían comer u ofrecían alimentos alternativos más saludables. El reconocimiento de haber fomentado el problema de los malos hábitos alimentarios y que no les dieron algunos alimentos para que los probaran desde pequeños^(24)^; refleja el dominio de experiencias pasadas que influyen de manera negativa en los hábitos actuales^(23)^. El control de emociones negativas, trascendental para el cuidado de las madres y componente de esencial atención antes de iniciar los proyectos de intervención, este cuidado garantiza un apego al tratamiento y éxito en las intervenciones^(27)^.

El autocontrol de los problemas emocionales, es valioso en el cuidado holístico en colaboración multidisciplinar, manifestado por el bienestar de que los niños comen lo mismo que los padres y son los padres quienes les enseñan a comer lo saludable y evitan mantenerlos en contacto con comida chatarra^(26)^, las madres estarían en posibilidad de que a partir de las preferencias saludables de los hijos, les podrían preparar los alimentos como ellos los prefieren para asegurar que los consuman y los disfruten. En la interacción de las madres con el entorno tecnológico y la práctica de la alimentación, se permite a los niños comer en la habitación y ver televisión porque es así como comen; caso contrario donde algunas madres luchan para que coman en la mesa y tienen un estilo disciplinado^(22)^. Algunos padres no autorizan que los hijos coman en el sofá o la habitación porque eso implica estar comiendo y viendo mientras comen. Un estudio mostró la importancia del rol de los padres quienes controlan la televisión, ellos son quienes la encienden y apagan, los hijos están condicionados y sólo les permiten ver televisión o usar teléfono cuando respetan las reglas, en caso contrario un castigo es no permitirles verlos, además son los padres los que deciden que programas pueden ver^(26)^. La tranquilidad de los padres porque los hijos comen “algo” es una decisión manipulada por la preocupación que disminuye en la disciplina y control de la permisión de ver televisión mientras los hijos comen. Asimismo, derivado de la interacción con el entorno simbólico con mayor disponibilidad de alimentos ultra procesados, las mujeres transitaron sus hábitos alimentarios^(28,29)^. Además, está demostrado que la tecnología influye en el consumo de alimentos hipercalóricos^(25)^.

Las madres trabajan y deben ajustar los horarios de preparación y consumo de alimentos de acuerdo con los horarios laborales, preparar comida rápida y fácil a pesar de que esta no sea saludable^(23)^; así en otro estudio, como consecuencia al exceso de trabajo las madres llegan a comprar comida preparada^(25)^. Además, hay coincidencias al tener restricciones de tiempo para preparar alimentos^(30)^, otros coinciden en que el exceso de trabajo de los padres no les permite preparar alimentos saludables y por la falta de tiempo necesitan comprar comida preparada fuera de casa^(25)^; agregando las preferencias y el gusto. Además, es una necesidad, delegar el cuidado de sus hijos a otros cuidadores familiares con quienes las madres comparten la crianza de los hijos particularmente durante el horario de trabajo, es durante este proceso cuando los hijos comparten los alimentos con cuidadores, así, las prácticas de crianza de alimentación influyen en los hábitos^(23,29)^. Lo anterior, vislumbra la necesidad de las intervenciones integrando cuidadores para fomentar hábitos alimentarios desde las familias.

Avanzar a nuevos hábitos de alimentación saludables, asequibles a los recursos económicos y culturalmente aceptables en cada familia, resolver brechas a través de políticas públicas para las mujeres jefas de vivienda, cero hambre, salud y bienestar para mujeres y niños. Finalmente, en las familias existen personas simbólicas como el esposo, los hallazgos de este estudio muestran la interacción de la madre con las preferencias y gustos alimentarios del esposo y la fuerte influencia sobre los significados que las mujeres atribuyen sobre lo que compran y cocinan para alimentar a todos los integrantes de la familia; lo que se muestra en los testimonios como la autoridad del padre y falta de autoridad como mujeres; asimismo, las creencias del padre sobre un cambio mágico en el peso de los hijos y que se quitará cuando los niños crezcan sin reconocer la obesidad como una enfermedad (Figura 2)^(25)^.

**Figura 2:** Modelo de los Significados que madres de niños con obesidad tienen sobre los hábitos alimentarios.

Fuente: Elaboración propia, 2023.

## LIMITACIONES DEL ESTUDIO

El análisis y colecta de datos tuvo interrupción debido a la variabilidad en los sistemas de organización de las instituciones educativas, las fechas de clases y vacaciones; a pesar de tomar en cuenta suspensiones y vacaciones se tuvieron dificultades de seguimiento a las entrevistas por períodos de suspensión internos.

Identificar conceptos sensibilizadores y escribir memos reflexivos sobre las experiencias personales y profesionales de los investigadores contribuyó a minimizar el sesgo; esta práctica permitió reconocer las propias creencias, conocimientos y experiencias, emociones y sentimientos, transformándolo en una oportunidad para profundizar en las conversaciones.

## CONCLUSIÓN

Las mujeres como seres sociales y personas holísticas cumplen diversos roles socialmente atribuidos como ser mujeres, esposas y madres; sin embargo, social, económica y culturalmente han evolucionado, trabajan y también realizan obligaciones en la crianza alimentaria de los hijos. Las madres al sentirse culpables por el tipo de alimentos que otorgan a sus hijos, los ceden porque es lo que, si comen, condescender ante el llanto y enojo de los hijos, quedarse en el intento de controlar el consumo de lo que ellas saben que no es saludable y por otra parte discutir porque quieren lograr que sus hijos coman verduras y obligarlos a comer lo que rechazan.

El modelo de los Significados que madres de niños con obesidad tienen sobre los hábitos alimentarios plantea una forma de valorar a las mujeres como seres sociales, una herramienta que guía la valoración holística como seres individuales y pertenecientes a un grupo social familiar. El cuidado de enfermería permite valorar y permitir expresar sus sentimientos, su sentir o preocupaciones; los testimonios muestran la evidencia científica de las realidades complejas de las mujeres que viven con respuestas humanas alteradas reconocidas como normales, como cuidadoras de sus hijos requieren planes de cuidados individuales considerando sus preferencias alimentarias, su cultura y realidades únicas; planear los cuidados familiares avanzando hacia un cuidado humanizado, reconociendo que los significados son un producto social que las madres forman como parte de una familia, grupos social, una cultural.

Se requiere un cambio de paradigma cultural desde el cuidado, fomentar la integración y obligatoriedad de los hombres a las intervenciones, dada la su importante contribución en la formación de hábitos alimentarios de mujeres e hijos, los roles de hombres y mujeres evolucionan, en la actualidad ambos padres trabajan y aportan a la economía, sin embargo, las actividades preparación de alimentos y otras que cultural y socialmente fueron atribuidas a mujeres deben evolucionar para contribuir a la sobrecarga de mujeres que además de trabajar realizan labores en el hogar y en la crianza de los hijos.

Desde la percepción de la mujer, las conductas y actitudes alimentarias de sus parejas son una fuerte influencia en la formación de los hábitos y comportamientos de sus hijos. Entre la regulación de los gustos y disfrute sobre las preferencias alimentarias del esposo, se vislumbra una oportunidad en el cuidado de enfermería al reconocer la importancia cultural y social del rol del padre como esposo, por lo tanto es necesario el padre de los niños estén informados y sean conscientes de la importancia comer y ofrecer una alimentación variada, equilibrada y saludable, lo que tendría impacto en su salud y la de sus hijos a lo largo de la vida, mejorando el bienestar y calidad de vida.
